# Decrease of Population Divergence in Eurasian Perch (*Perca fluviatilis*) in Browning Waters: Role of Fatty Acids and Foraging Efficiency

**DOI:** 10.1371/journal.pone.0162470

**Published:** 2016-09-09

**Authors:** Kristin Scharnweber, Ursula Strandberg, Konrad Karlsson, Peter Eklöv

**Affiliations:** 1 Uppsala University, Evolutionary Biology Centre, Department of Ecology and Genetics; Limnology, Uppsala, Sweden; 2 University of Eastern Finland, Department of Biology, Joensuu, Finland; 3 Stockholm University, Department of Ecology, Environment and Plant Sciences, Stockholm, Sweden; University of Hyogo, JAPAN

## Abstract

Due to altered biogeochemical processes related to climate change, highly colored dissolved organic carbon (DOC) from terrestrial sources will lead to a water “brownification” in many freshwater systems of the Northern Hemisphere. This will create deteriorated visual conditions that have been found to affect habitat-specific morphological variations in Eurasian perch (*Perca fluviatilis*) in a previous study. So far, potential drivers and ultimate causes of these findings have not been identified. We conducted a field study to investigate the connection between morphological divergence and polyunsaturated fatty acid (PUFA) composition of perch from six lakes across a gradient of DOC concentration. We expected a decrease in the prevalence of PUFAs, which are important for perch growth and divergence with increasing DOC concentrations, due to the restructuring effects of DOC on aquatic food webs. In general, rate of morphological divergence in perch decreased with increasing DOC concentrations. Proportions of specific PUFAs (22:6n-3, 18:3n-3, 20:5n-3, and 20:4n-6) identified to primarily contribute to overall differences between perch caught in clear and brown-water lakes tended to be connected to overall decline of morphological divergence. However, no overall significant relationship was found, indicating no severe limitation of essential fatty acids for perch inhabiting brown water lakes. We further broaden our approach by conducting a laboratory experiment on foraging efficiency of perch. Therefore, we induced pelagic and littoral phenotypes by differences in habitat-structure and feeding mode and recorded attack rate in a feeding experiment. Generally, fish were less efficient in foraging on littoral prey (Ephemeroptera) when visual conditions were degraded by brown water color. We concluded that browning water may have a strong effect on the forager’s ability to find particular food resources, resulting in the reduced development of evolutionary traits, such as habitat- specific morphological divergence.

## Introduction

In the face of rapid human-induced environmental changes the ability of organisms to successfully survive and reproduce is challenged, and is primarily determined by their ability to adaptively respond to these changes [1]. Environmental variations will influence community interactions, alter the degree of individual resource specialization and cause displacement in the organisms’ niches [2]. This will have further effects on the development of evolutionary important traits related to resource use, potentially leading to disruptive selection [3].

Lakes provide distinct habitats where fish segregate by diet, leading to an early stage of population divergence [4, 5]. When differences in resource use are stable over time, natural selection may lead to morphological adaptations and polymorphism [6, 7]. An on-going homogenization, or flattening of ecological gradients of the environment due to human alteration may weaken the effect of divergent selection [8, 9], potentially leading to reverse speciation [10, 11]. Currently, there are strong changes in aquatic ecosystems due to altered human activities [12]. In particular, water transparency is affected by numerous factors, such as eutrophication [13] or elevated sediment loadings [14].

In recent years, a strong increase in the flux of dissolved organic carbon (DOC) into aquatic systems has been identified as being due to climatic factors [15–17]. This phenomenon, dubbed “brownification”, is strongly and negatively correlated to light transmission [18, 19]. Brownification may cause a homogenization effect as a deteriorated light regime may decrease macrophyte growth, which will reduce the spatial dimension of the near-shore littoral zone [20] and impede the growth of benthic algae, resulting in a stronger reliance on pelagic (open-water) pathways [21]. These effects will erase habitat distinctions between littoral and pelagic zones. So far, ongoing research has mainly focused on the effects of increasing DOC concentration on the microbial food web and its implication for carbon sequestration (e.g. [22, 23]). However, very little is known about the effects of increasing DOC-concentration on the higher trophic level organisms of the food web (but see [24, 25]) and especially on the consequences for population divergence and the evolution of organisms.

In this study, we investigated the population divergence of Eurasian perch (*Perca fluviatilis*), in relation to food quality. Perch is a common predator in boreal lakes that shows strong, habitat-specific morphological adaptations [26, 27]. In perch, foraging in the open water is associated with a high search rate for widely distributed and conspicuous planktonic prey, favoring a more streamlined body [26, 27]. In contrast, when foraging in the structurally complex near–shore zone perch has a lower search rate for the more cryptic benthic prey, thus favoring a deeper body form [26, 27]. In a previous study, population divergence of perch was found to be low in low water transparency, and the overall reliance on littoral resources decreased [28, 29]. However, the ultimate causes and underlying mechanism for this pattern remained unclear. Perch growth is constrained by nutrient stoichiometry (ratios of carbon, nitrogen, and phosphorus) of whole perch, and perch show strong intraspecific variations in stoichiometry related to both morphological and dietary specialization [30]. Furthermore, the biogeochemical compositions of resources, for example fatty acid (FA) composition is known to be essential for fish growth [31, 32]. Therefore, food quality and the forager’s ability to choose among food items of different qualities should be major factors contributing to differences in population divergence.

Light limitation by DOC has an effect on secondary production and the trophic transfer to top predators [17, 33]. First of all, DOC may alter the overall structure of food webs due to shading effects on autotrophs, instead stimulating growth of heterotrophic bacteria [23, 34]. Karlsson et al. [18] found decreased growth of benthic production due to light limitation that even can translate to lower fish production (see also [25]). Along the same line, Finstad et al. [24] showed that high DOC concentration limit the biomass of brown trout (*Salmo trutta*) in shallow lakes, which was assumed to be connected to the overall reconstruction of food webs due to light limitations. In addition, DOC may negatively affect the overall availability of essential biogeochemical compounds within the aquatic food web due to a change of phytoplankton community structure [35–37]. Phytoplankton is the major source of polyunsaturated fatty acids (PUFAs) for consumers in freshwater ecosystems [38, 39] which are essential to support optimal health in consumers [40]. Especially n-3 and n-6 PUFAs which have double bonds at the third, such as 20:5n-3 and 22:6n-3 or sixth location from the methyl end bonds, e.g. 20:4n-6, have been found to play an important role in growth and reproduction in aquatic animals [32, 39]. In previous studies, variation of FA composition was found to be connected to increasing DOC concentrations in pelagic zooplankton [36], but also in the common littoral invertebrate *Asellus asellus* [41], with lower proportions of PUFAs in high DOC lakes. These results imply that perch from lakes of high DOC concentration may consume prey of low PUFA content, resulting in inefficient energy transfer, potentially leading to impaired growth and divergence. Empirical studies from the field that investigate the effects of resources of differing quality to the production of fish are scarce (but see [42, 43]). However, Litzow et al. [44] demonstrated a connection between lipid content in fish communities in boreal oceans and species abundances. They postulated an “essential fatty acid limitation hypothesis”, suggesting a direct connection between fish production and the availability of essential FAs [44].

In addition to the overall restructuring effect of DOC at the base of the food web, light limitation by DOC may further impair efficiency of visual foraging of fish [45]. Therefore, resource use in brown waters can be altered due to the change of perception of prey and decreased reaction distance [46, 47]. Similarly, to the effects caused by turbidity, this may have consequences for the selection of preferred or optimal prey in relation to food quality [48, 49]. Therefore, altered foraging efficiency on specific prey items due to deteriorated visual conditions may also be an underlying mechanism for a decrease in population divergence of perch.

In this study, we examined the effects of DOC on population divergence of perch using two different approaches. First, we evaluated the general hypothesis that an increase of DOC concentration will reduce the abundance of PUFAs in perch due to the underlying restructuring effects of DOC on aquatic food webs. As PUFAs are essential for fish growth and growth rate in perch is directly related to phenotypic development and the rate of divergence, we predict lower proportions of essential FAs in perch of high DOC lakes, where population divergence was found to be low. Alternatively, perch divergence can be expected to be low due to difficulties in finding specific prey under deteriorated visual conditions. Therefore, we augment our dataset by measuring foraging efficiency on pelagic and littoral prey items in a controlled laboratory experiment. In accordance to the findings of Bartels et al. [29], who demonstrated a decrease in the reliance on littoral resources with increasing DOC concentrations, we predict a lower efficiency in foraging on littoral prey in brown water conditions.

## Materials and Methods

### Field study

#### Study areas and sampling

**Fig 1:**
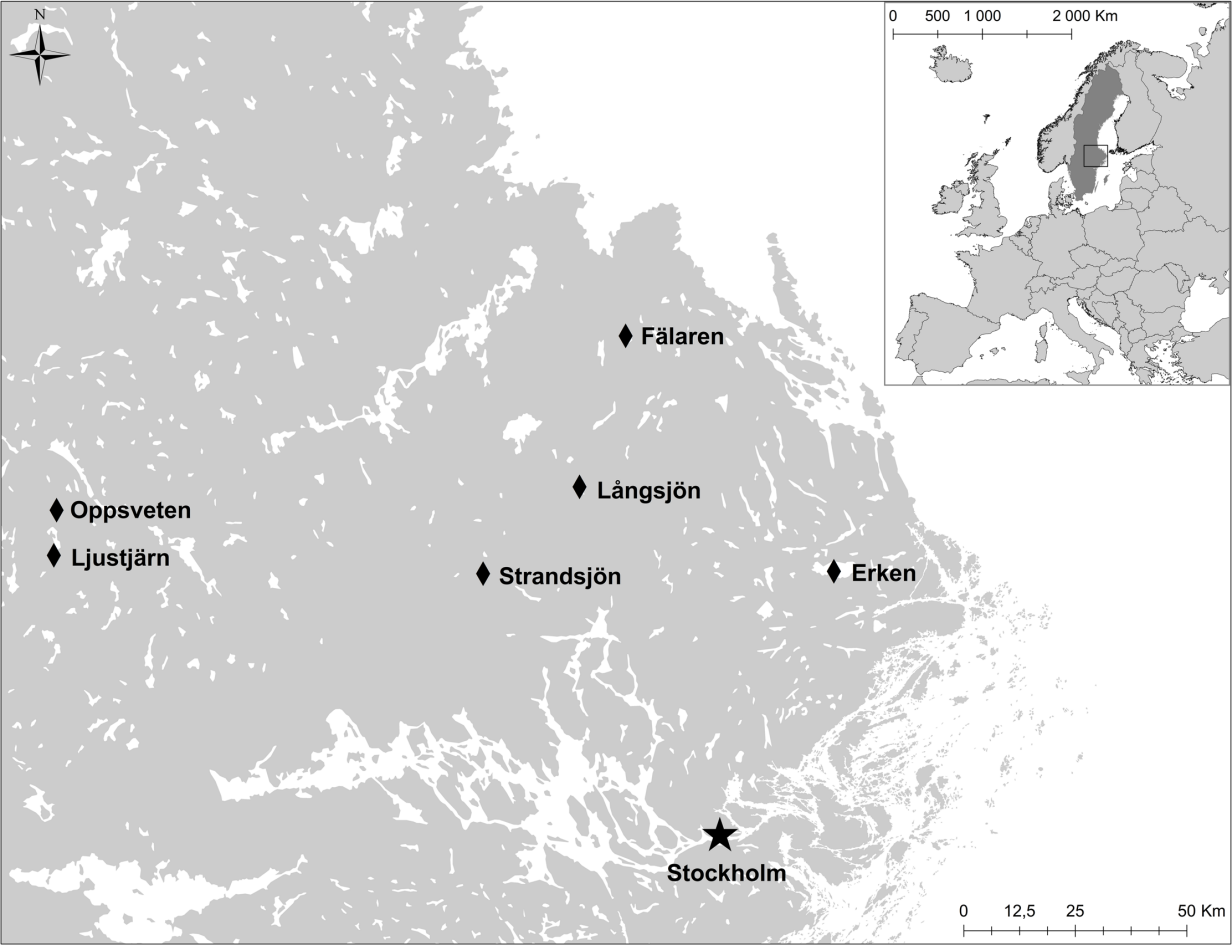
Locations of the six lakes surveyed in this study. Copyright by Lantmäteriet Gävle (2012): Permission i2012/921.

To study the effects of fatty acid composition on population divergence of perch, we conducted a field survey across six lakes located in Central Sweden (Fig 1).

Necessary permits were obtained from the land owners and respective authorities. Three of the lakes can be characterized as clear-water lakes with a secchi depth (commonly used as a measure to describe water transparency) of more than 2.5m and low DOC concentrations (below 11mg l ^-1^) and three lakes were brown-water lakes with a secchi depth below 2.0m and high DOC concentrations (more than 13mg l ^-1^; Table 1).

**Table 1:** Main characteristics of the surveyed lakes in Central Sweden.

	Ljustjärn	Långsjön	Erken	Oppsveten	Strandsjön	Fälaren
Overall characteristic	Clear-water	Clear-water	Clear-water	Brown-water	Brown-water	Brown-water
Location	N59°54´ E15°23´	N60°01´ E17°34´	N59°50´ E18°33´	N60°00´ E15°25´	N59°52´ E17°09´	N60°20´ E17°47´
Area (km^2^) ^1^	0.12	2.50	23.70	0.65	1.30	2.05
Max depth (m) ^1^	11.0	12.5	21.0	10.0	4.0	2.6
Dissolved organic carbon (mg l ^-1^) ^2^	3.0±<0.1	6.0±0.2	10.9^3^	13.1±0.2	15.9±0.4	25.5±0.2
Total phosphorus (μg l ^-1^) ^2^	7.1±1.6	21.1±1.4	27.2±11.8	10.4±1.1	70.8±3.8	28.8±0.3
Secchi depth (m) ^4^	6.2	2.9	3.4	1.8	0.9	0.8
*N* perch caught (littoral/pelagic)	13/21	19/7	37/36	20/11	18/17	7/6

^1^ values were adapted from Bartels et al. [29]

^2^ values represent average values (± standard deviation) of three measurements at 1m depth, taken in August 2014

^3^ measurement was taken in June 2014

^4^ measurement was taken in August 2014

Trophic state of the lakes ranged from oligotrophic to eutrophic and DOC was highly correlated to total phosphorus (TP) concentrations (Spearman´s rank correlation (r_s_) = 0.771; *P* < 0.0001), demonstrating the linkage between terrestrial carbon concentration and nutrients in freshwater systems (see also Bartels et al. [29] on further information on the lakes). In August and September 2014, fish were caught using multi-mesh gill nets (littoral nets: 30 x 1.5m; pelagic nets: 27.5 x 6m). For up to 12 hours, nets were set at shallow, near-shore (littoral) and open-water (pelagic) zones (see Table 1 for the number of perch caught in the respective habitats). We used only fresh fish with red gills for fatty acid analysis and fish were stored on ice during the transport to the laboratory where they were transferred to -20°C. In the laboratory, fish were partially thawed and measured to the nearest 1mm (total length) and weighed to the nearest 0.1g. As perch are known to undergo a ontogenetic niche shift from being pelagic zooplanktivorous as juveniles, then being omnivorous and including benthic invertebrates in the diet at intermediate size classes, and later becoming piscivorous at large size classes [50], we targeted individuals within the intermediate size spectra (total length of 114.5mm ± 17.8mm SD) that are known feed on both invertebrates and zooplankton (e.g.[50]). A photograph was taken of the left side of the fish with fins stretched out. A piece of dorsal muscle tissue was taken that was kept at -20°C for subsequent fatty acid analysis.

#### Geometric morphometrics

We estimated perch morphology by using a landmark-based geometric morphometrics [51]. Digital photographs were transferred to TPSdig2 (http://life.bio.sunysb.edu/morph/) and 16 landmarks were digitized *sensu* Bartels et al. [29] and Scharnweber et al. [52]. Variation in morphology between littoral and pelagic perch of the six lakes were assessed using MorphoJ [53]. We checked for outliers using the “Find outliers” function. A regression of the normally distributed shape scores (Procrustes coordinates) on size (centroid size) for each lake were used separately to correct the shape data for body size [54]. Residuals obtained from this regression were used for all further analysis. To assess significance of shape differences (Mahalanobis distances, MD) between littoral and pelagic perch, we used Discriminant Function Analyses (DFA). We performed nonparametric Spearman’s rank correlations tests using IBM SPSS Statistics 21 (IBM Corporation, Armonk, NY, USA) to evaluate the connection of morphological divergence and DOC concentrations.

#### Fatty acid analysis

To analyze FA compositions of perch and resources, we followed the approach described in Scharnweber et al. [52]. Approximately 200mg of fresh muscle tissue was dissected. Lipid extraction was conducted in chloroform/methanol (2:1, by volume) and 0.88% KCl was added to remove non-lipids. After vortexing and centrifuging, extraction procedure was repeated and both organic phases were pooled. To enhance the extraction a sonification of 10min was used and total lipid extracts were concentrated under a nitrogen stream. Lipids were dissolved in hexane and transmethylated at 90°C for 90min, using acidic catalyst (1% H_2_SO_4_ in Methanol).

Analyses of fatty acid methyl esters (FAME) were conducted at the University of Eastern Finland using an Agilent 6890 N Gas Chromatographer (Agilent Technologies, Santa Clara, CA, USA) equipped with a DB-23 column (length 30m, ID 0.25mm, film thickness 0.25μm, Agilent). A split injection (20:1) was applied, using an initial temperature of 180°C for 8min, which then was increased by 2°C min^-1^ to 210°C, and was finally maintained for 2 min. Helium gas was used as a carrier with an average velocity 36cm sec^-1^.

FAME peaks were identified using retention times and mass spectra using the software MSD ChemStation (F01.01.2317; Agilent Technologies). A heneicosanoic acid (Nu-Chek Prep, Inc. Elysian, MN, USA) was used as an internal standard. Using calibration curves of standard solutions of known lipid mixtures (Nu-Chek Prep, Inc.) we calculated FA concentrations, and data was expressed as percentages relative to total FA (FA %). Multivariate analyses were performed using Primer 7.0.6 with the PERMANOVA add-on (Primer E Ltd. Plymouth, United Kingdom). We arcsine-square root transformed fatty acids (% fatty acids) and based ordination on Euclidean distance matrices. PERMANOVA, the non-parametric analogue of MANOVA was used to test FA differences between lakes, habitats (nested within lake), and total length was used as a covariate. To determine significance of analyses we conducted permutation of residuals under a reduced model (9999 permutations) with type I sums of squares [55]. Proportion of variance explained was calculated from sums of squares. We further used distance-based redundancy analyses (DistLM) to investigate the connection between fatty acid composition and environmental factors that were not categorical. As PERMANOVA; DistLM uses a “semiparametric” approach. Therefore, there were no constriction to particular distributions of the data [55]. Model parameters were set with r^2^ as a selection criterion and 9999 permutations. To depict variation of fatty acid composition of fish we carried out non-metric multidimensional scaling (nMDS). Fatty acids that contributed most to the observed differences between perch caught in clear- and brown-water lakes were identified using the similarity percentages routine (SIMPER).

### Laboratory experiment

#### Fish collection and induction of phenotypes

To study the effects of brownification on foraging efficiency of perch phenotypes, we conducted a laboratory experiment. The experiments were carried out in strict accordance with the recommendations of the ethical committee of Uppsala Djurförsöksetiska Nämnden (Permit number C231/10). Fish were sacrificed using an overdose of fenoxy ethanol, and all efforts were taken to minimize suffering.

In July 2011, young-of-the-year fish from Lake Erken, which has been surveyed in the field study, were captured using a seine net, and transported to the holding facility at the Swedish Board Fisheries laboratory at Drottningholm, Stockholm. Littoral and pelagic phenotypes were induced by differences in habitat structure and feeding mode following the approach of Olsson and Eklöv [56]. Captured fish were held in two cylindrical 7m^3^ tanks fed by filtered lake water at densities of about 60–70 individuals per tank. These tanks either resembled the unstructured pelagic habitat where the tank received no physical structure, or the structurally complex littoral habitat where the tank received artificial structure consisting of plastic strings (300 strings m^2–1^) attached to an iron grid and placed at the bottom. Both populations obtained the same amount of food (Chironomidae of 15% individual wet weight−^day^), but feeding modes differed between the two holding tanks. Fish in the littoral tank were fed from the bottom, therefore, Chironomidae were placed on squares of plastic turf that were lowered to the bottom by using a platform. In contrast, fish in the pelagic tank were fed by spreading the food at the surface. After 8 weeks, fish were transported from the holding facility to Uppsala University where the experiments were conducted.

#### Experimental set-up and data analysis

Experiments were conducted in 30l –tanks (50 x 25 x 25cm) with the bottom covered by a 3cm thick layer of sand. As perch are social foragers [57], three individuals of the same phenotype were used in each replicate. Water color was manipulated by using a commercially available brown water conditioner (Sera Blackwater Aquatan, Sera GmbH, Heinsberg, Germany) that consist of humic matter but does not have any effect on the water pH. Within the brown water tanks, light level was reduced by the colored water to 8.52μmol ± 0.34 standard error (SE) compared to 48.77μmol ± 2.04 SE (measured at the bottom of the tank). Fish were fed by a mix of live food consisting of pelagic (*Daphnia* sp.; 275 items per replicate) and littoral prey items (Ephemeroptera; 20 items per replicate), but foraging rate was recorded for either one of the items. By this, any other activity than foraging on *Daphnia* (such as foraging on Ephemeroptera) was reflected in a lower foraging rate, and *vice versa*. The experimental design was a 2 x 2 full factorial (replicated 8 times) with two levels of phenotype (littoral, pelagic) and two levels of visual condition (clear, brown) for each of the feeding treatments (focus on *Daphnia*-foraging, focus on Ephemeroptera-foraging). To estimate foraging rate on *Daphnia*, time to capture 30 prey items per fish was measured. Within the trials to estimate foraging rate on Ephemeroptera, time to capture 12 items was measured. As *Daphnia* represented very small prey items that had to be consumed in high numbers, individual average values for each of the three fishes were taken within each of the trials with a focus on foraging rate of pelagic prey. In contrast, Ephemeroptera represented a much larger prey item and therefore fewer prey items have to be consumed before satiation. Therefore, time for all three fish to capture 12 prey items were taken within the trials with a focus on foraging rate of littoral prey. Fish were starved for 24 hours to ensure that they were motivated to feed. All trials were video recorded and analyzed later-on. Analyses were performed using R 3.2.3 [58], and the package lme4 [59]. For the analysis of littoral foraging on Ephemeroptera, we used a linear model, but for the analyses of pelagic foraging on *Daphnia*, there was an unequal number of observations within each trial (replicate) and we therefore used a generalized mixed model instead, as suggested by Bolker et al. [60]. Data from each individual fish was used as response, and phenotype, visual condition, and their interaction was used as fixed effects. For the analysis of pelagic foraging on *Daphnia*, we accounted for variation within replicate by adding replicate as random effect and *P*-values were rendered by normal distribution approximation [61].

After ending the experiment, photographs were taken from the left side of 30 randomly chosen individuals of each treatment and perch morphology was assessed using the geometric morphometric approach as described above.

## Results

### Field study

**Fig 2:**
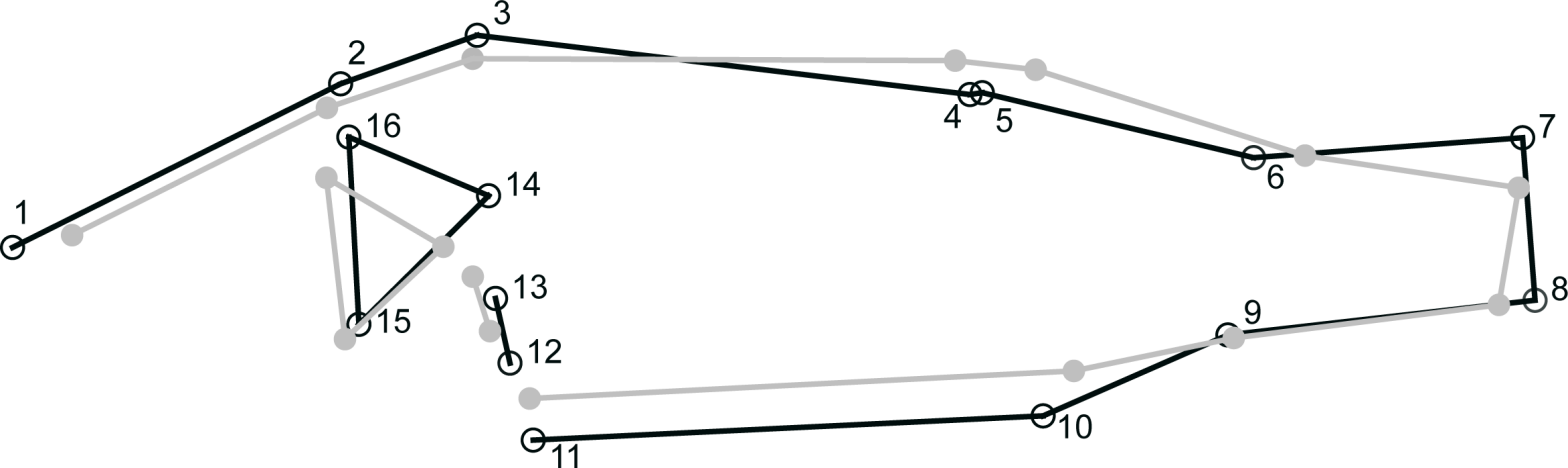
Results of shape analyses of perch caught in the field. Shape differences between littoral (black line) and pelagic (grey line) perch of the six lakes studied. Positions of the 16 digitized landmarks used in geometric morphometrics are shown. Shape-change outlines of Discriminate Function Analysis are magnified ten-fold.

**Fig 3:**
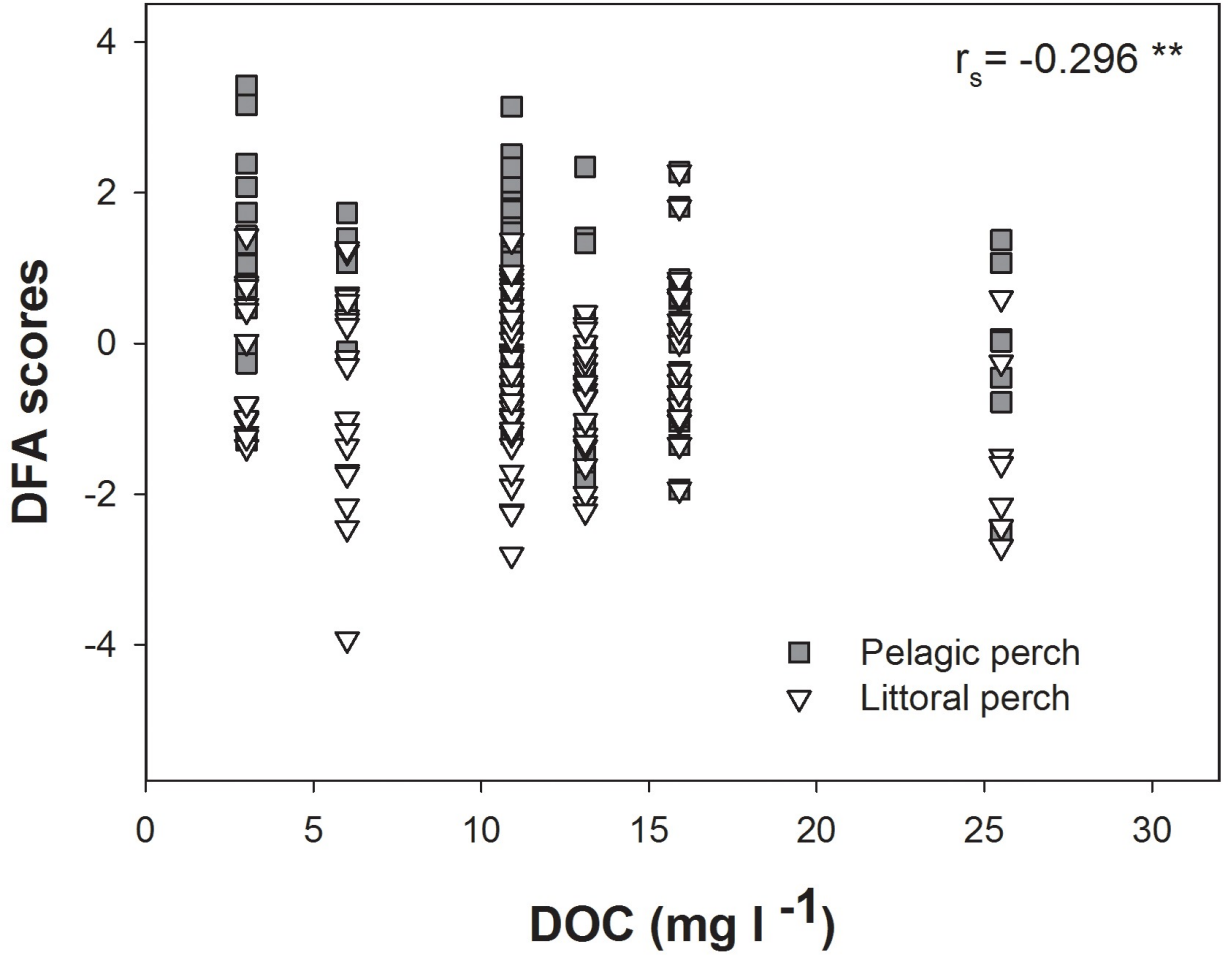
Connection between shape and DOC concentrations. Correlation between scores of Discriminant Function Analysis between perch caught in the littoral and pelagic zone and DOC concentrations. Spearman’s rank correlation coefficient is shown. ** *P*<0.005.

We estimated perch morphology for 212 individual perch. Overall, there was a significant difference in shape between perch caught in the littoral and pelagic zone across all lakes (DFA; MD = 1.1382, *P* < 0.0001) with individuals from the littoral zone having a deeper body compared to individuals from the pelagic zone that were more streamlined (Fig 2). Scores of DFA were negatively correlated to DOC concentration (r_s_ = -0.296; *P* < 0.0001; Fig 3) illustrating a moderate, but significant decrease of divergence with increasing DOC concentrations.

From each habitat and lake we analyzed fatty acids of six individual perch (*N* = 72). In total, we identified 37 fatty acids (Table 2). Fatty acid composition differed significantly between perch caught in the littoral and pelagic zones (PERMANOVA: Pseudo-*F* = 2.98; *P* = 0.0001). The factor habitat nested within lakes explained 15.0% of the variance of fatty acid composition. Furthermore, results from PERMANOVA showed a significant difference between perch from the six studied lakes (Pseudo-*F* = 7.45; *P* = 0.0001, 31.2% of variance). Total length as a covariate had also a significant effect on fatty acid composition (PERMANOVA: Pseudo-*F* = 5.21; *P* = 0.0013). Results were similar when analyses were repeated for clear- and brown-water lakes separately (S1 Table). DistLM showed a significant relationship between DOC and fatty acid composition across the whole dataset (Pseudo-*F* = 3.44; *P* = 0.0079), and 4.7% of the variance was explained by DOC.

**Table 2:** Fatty acid composition (%) perch caught in six lakes in Central Sweden.

Fatty acid (%)	Ljüstjarn		Långsjön		Erken		Oppsveten		Strandsjön		Fälaren	
	littoral	pelagic	littoral	pelagic	littoral	pelagic	littoral	pelagic	littoral	pelagic	littoral	pelagic
14:0	1.4±0.4	1.3±0.1	0.6±0.3	0.9±0.1	0.9±0.2	1.3±0.2	0.6±0.2	1.1±0.3	0.9±0.3	1.1±0.3	0.7±0.3	0.8±0.2
i-15:0	0.2±<0.1	0.1±<0.1	0.1±<0.1	0.1±<0.1	0.2±0.1	0.3±0.1	0.1±<0.1	0.1±<0.1	0.2±0.1	0.2±0.1	0.1±<0.1	0.1±<0.1
15:0	0.4±<0.1	0.4±<0.1	0.3±0.1	0.3±0.1	0.4±0.1	0.4±<0.1	0.3±0.1	0.5±0.2	0.4±0.1	0.5±<0.1	0.6±0.1	0.8±0.1
i-16:0	0.3±0.2	0.4±0.1	1.0±0.4	0.6±<0.1	0.7±0.2	0.4±0.2	0.5±0.1	0.5±0.3	0.7±0.1	0.7±0.1	0.7±0.3	0.7±0.3
16:0	19.3±1.0	20.1±0.6	22.8±1.5	23.0±1.2	21.7±0.8	21.6±1.0	22.0±0.5	20.7±0.9	21.1±0.8	21.1±0.5	21.6±0.8	21.3±0.8
16:1	0.3±0.1	0.3±<0.1	0.2±0.1	0.2±0.1	0.2±<0.1	0.3±<0.1	0.2±<0.1	0.3±<0.1	0.2±0.1	0.3±0.1	0.3±0.1	0.2±0.1
16:1n-9	0.3±0.1	0.4±0.1	0.4±0.1	0.5±0.1	0.4±0.2	0.7±0.1	0.3±<0.1	0.4±0.1	0.5±0.1	0.5±0.1	0.4±0.1	0.5±0.1
**16:1n-7**	1.2±0.8	0.7±0.1	2.0±0.7	1.2±0.3	3.8±1.7	2.6±0.5	2.0±0.9	1.8±0.3	2.6±0.7	2.5±0.8	2.1±0.9	2.0±0.8
16:1n-5	0.5±0.1	0.5±<0.1	0.3±0.1	0.4±0.1	0.4±<0.1	0.4±<0.1	0.5±0.1	0.5±0.1	0.5±0.1	0.5±0.1	0.5±0.1	0.5±0.1
i-17:0	0.3±0.1	0.3±<0.1	0.2±0.1	0.2±0.1	0.2±0.1	0.3±<0.1	0.1±<0.1	0.2±<0.1	0.3±0.2	0.3±<0.1	0.2±0.1	0.2±0.1
ai-17:0	0.2±0.1	0.2±<0.1	0.1±0.1	0.1±<0.1	0.1±<0.1	0.1±<0.1	0.1±<0.1	0.2±<0.1	0.2±0.1	0.2±<0.1	0.1±0.1	0.1±<0.1
17:0	0.7±0.3	0.7±0.1	0.5±0.1	0.6±0.1	0.7±0.1	0.7±<0.1	0.5±0.1	0.6±0.2	1.0±0.2	1.0±0.1	0.7±0.1	1.0±0.1
17:1	0.2±0.1	0.1±<0.1	0.2±<0.1	0.1±<0.1	0.2±0.1	0.2±<0.1	0.1±0.1	0.2±0.1	0.3±0.2	0.3±0.1	0.3±0.1	0.4±0.1
18:0	7.1±0.3	7.2±0.1	8.2±0.8	7.2±0.4	7.4±0.6	6.6±0.4	7.4±0.2	7.1±1.0	7.6±0.2	7.4±0.4	7.4±0.7	7.9±0.4
18:1n-9	5.0±0.6	4.7±0.6	5.6±0.9	6.1±0.5	5.2±1.0	6.1±0.5	4.0±0.5	6.1±0.7	5.4±1.1	4.7±0.7	6.1±3.2	4.9±0.9
18:1n-7	2.7±0.8	2.7±0.3	2.8±0.6	2.5±0.2	4.3±0.7	3.2±0.2	3.5±0.3	3.1±0.3	3.1±0.8	2.5±0.7	2.8±0.4	3.2±0.9
18:2n-6	2.0±1.3	1.2±0.2	2.3±1.1	2.6±0.5	2.7±0.6	2.3±0.2	1.8±0.4	1.9±0.2	2.9±1.1	1.9±0.9	1.6±0.4	2.4±0.9
**18:3n-3**	1.6±0.4	1.2±0.2	0.4±0.2	1.7±0.5	1.6±0.5	3.0±0.5	0.9±0.2	1.1±0.3	3.6±2.2	2.3±1.6	0.7±0.3	0.9±0.3
18:4n-3	0.9±0.4	0.6±0.1	0.1±0.1	0.4±0.2	0.5±0.2	1.2±0.2	0.4±0.3	0.8±0.4	0.5±0.3	0.5±0.2	0.4±0.4	0.3±0.1
**20:4n-6**	9.4±2.2	12.1±1.6	9.5±0.6	10.8±0.9	7.4±1.2	8.1±0.5	12.2±1.0	10.0±0.9	9.1±2.6	8.5±1.3	11.0±1.2	11.1±1.4
20:4n-3	0.2±<0.1	0.2±<0.1	0.1±0.1	0.1±<0.1	0.2±0.1	0.4±0.1	0.1±<0.1	0.2±0.1	0.2±0.1	0.3±<0.1	0.2±0.1	0.2±<0.1
**20:5n-3**	11.2±1.8	14.0±1.4	11.2±2.8	14.8±1.9	14.4±1.3	14.2±0.7	11.8±1.8	10.0±1.4	12.0±1.4	11.7±0.9	10.0±1.7	10.7±2.8
22:4n-6	0.5±0.2	0.6±0.2	0.5±0.2	0.4±<0.1	0.4±0.1	0.5±0.1	0.5±0.1	0.5±0.3	0.4±0.1	0.3±0.2	0.6±0.1	0.7±0.1
22:5n-6	4.2±1.4	2.6±0.5	1.9±0.5	1.7±0.3	1.5±0.2	1.6±0.1	2.5±0.3	3.1±0.3	2.2±0.5	2.6±0.6	2.8±0.5	2.6±0.3
22:5n-3	1.4±0.6	1.9±0.5	2.6±0.7	2.0±0.5	2.3±0.3	2.1±0.3	1.7±0.3	1.3±0.4	1.7±0.4	1.6±0.4	1.9±0.4	2.3±0.6
**22:6n-3**	26.8±5.9	24.2±4.0	24.6±5.3	20.1±1.6	20.6±2.2	19.6±2.3	24.3±1.4	25.8±2.9	20.4±4.1	24.9±3.9	24.1±4.0	22.2±5.1
24:1n-9	1.0±0.2	0.8±0.1	1.0±0.2	0.7±0.1	0.9±0.1	1.0±0.1	1.0±0.1	1.1±0.3	0.8±0.3	1.0±0.2	1.4±0.2	1.1±0.4
∑ SFA	30.2±0.7	30.8±0.3	34.0±1.3	33.2±0.8	32.6±0.8	32.2±1.0	31.9±0.5	31.4±2.1	32.7±0.8	32.7±0.4	32.5±1.4	33.4±1.0
∑ MUFA	11.3±2.0	10.3±1.1	12.5±1.7	11.9±1.1	15.6±2.9	14.6±1.3	11.7±1.2	13.7±1.1	13.8±2.5	12.3±1.6	14.0±3.9	13.1±1.6
∑ PUFA	58.4±2.4	58.8±1.0	53.4±2.0	54.8±0.9	51.9±2.2	53.2±1.1	56.4±1.1	54.9±2.6	53.5±2.8	55.0±1.5	53.5±3.6	53.6±2.0
∑ n-6	16.3±1.7	16.6±1.3	14.4±1.0	15.6±0.6	12.3±0.9	12.6±0.5	17.1±1.0	15.7±0.6	14.2±0.9	13.6±1.0	16.2±1.1	17.0±1.4
∑ n-3	42.1±4.1	42.2±2.0	39.1±2.4	39.3±1.2	39.6±2.4	40.6±1.3	39.3±2.1	39.2±3.1	38.1±3.2	41.4±2.1	37.3±3.2	36.6±2.3
∑ Bacterial	2.5±0.2	2.3±0.3	2.4±0.6	2.1±0.4	2.6±0.4	2.7±0.4	1.8±0.2	2.5±0.8	3.7±0.9	3.2±0.3	2.9±0.5	3.5±0.4
n-3/n-6	2.6±0.5	2.6±0.3	2.7±0.3	2.5±0.1	3.3±0.4	3.2±0.2	2.3±0.3	2.5±0.3	2.7±0.4	3.1±0.3	2.3±0.2	2.2±0.3

Averages of six individual perch analyzed from each habitat and lake ± SDs (Total *N* = 72) are shown. Only those FA are presented that account for > 0.3% at least in one group. Sums (∑) of saturated fatty acids (SFA) list fatty acids without a double bond, excluding branched and odd chained saturated fatty acids. Sums of mono-unsaturated fatty acids (MUFA) list fatty acids with a single bond, and sums of polyunsaturated fatty acids (PUFAs) list fatty acids with more than one double bond. Iso- and anteiso- branched and odd chained saturated fatty acids are summarized as bacterial fatty acids. Bold font depict fatty acids that contribute most to differences between perch caught in clear- and brown-water lakes.

**Fig 4:**
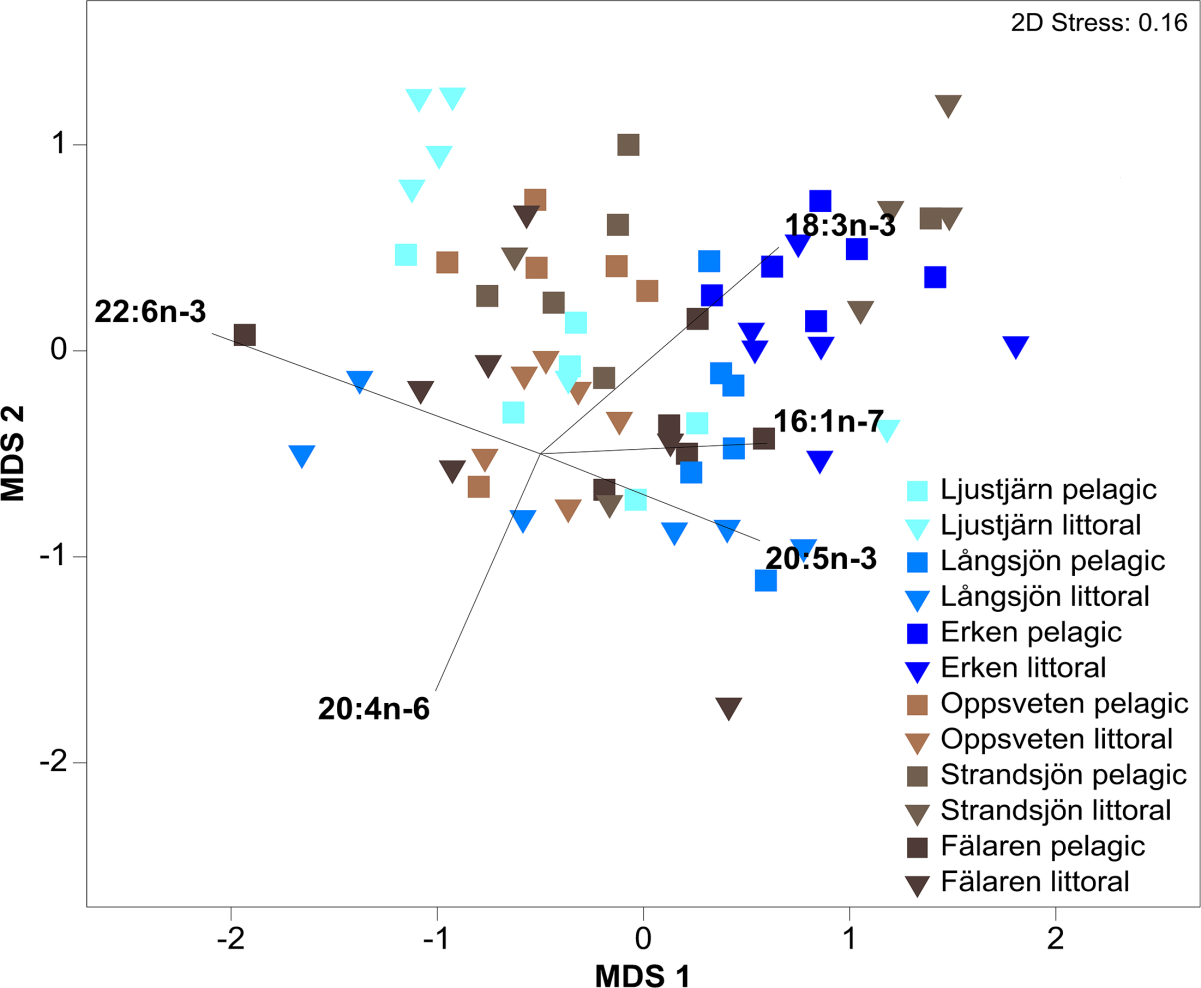
2D nMDS of fatty acid composition. Two-dimensional representation of non-metric multidimensional scaling (nMDS) of fatty acid composition (%) of perch caught in the littoral and pelagic zone of the six lakes surveyed. Color shading illustrates the gradient of DOC from light blue = low DOC to dark brown = high DOC. Relative length of vectors from fatty acids identified to contribute most to observed difference in composition between perch caught in clear-and brown water-lakes depict strength in positioning in the respective dimension.

NMDS visualized the observed pattern in fatty acid composition in perch across the six lakes (Fig 4). The stress-level of 0.16 in the two-dimensional nMDS indicated a “good” fit of ordination into the multidimensional space [55]. However, we further refer to an animation in the three-dimensional space (stress-level 0.09) for a better inspection of the data (see video in S1 File).

**Fig 5:**
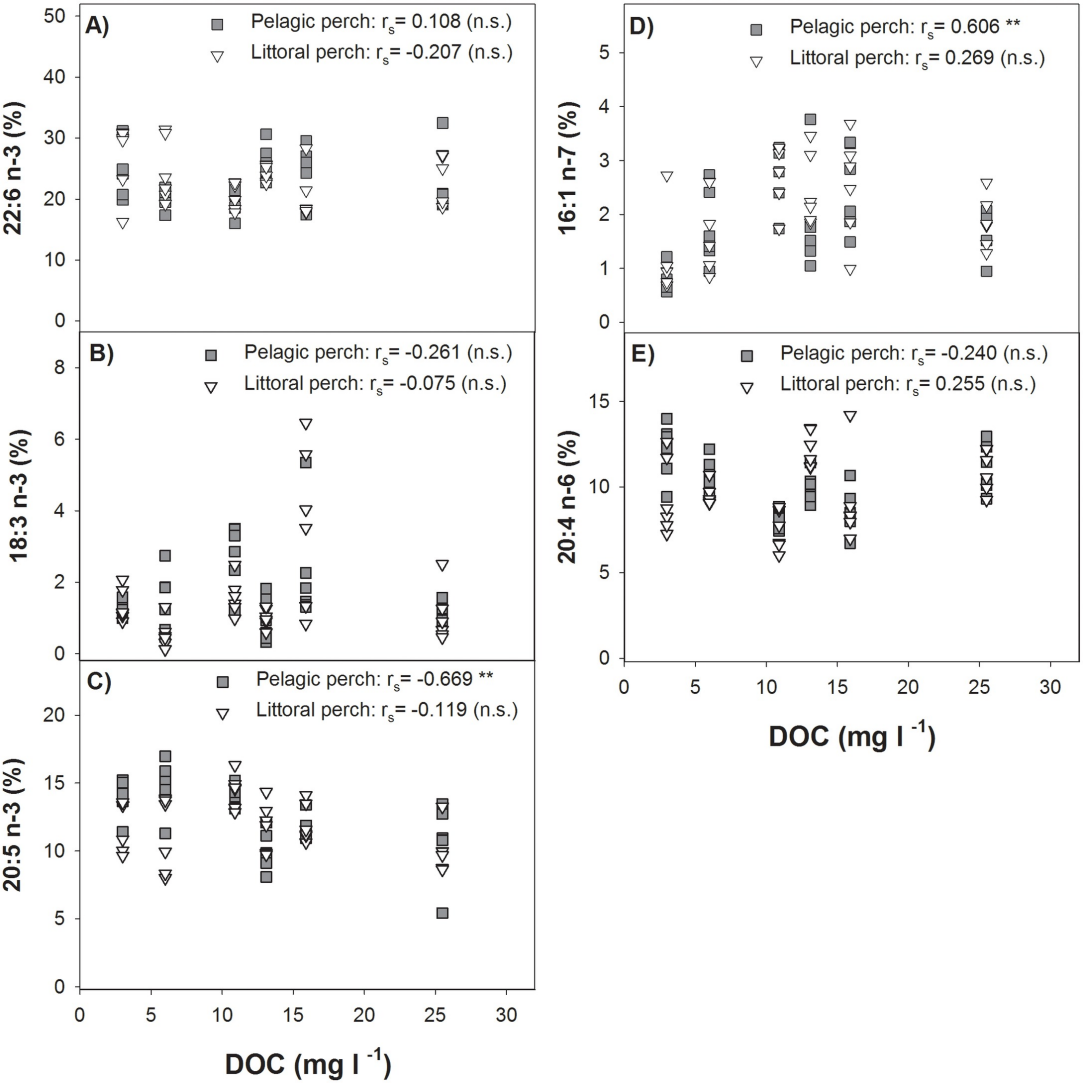
Connection between fatty acids and DOC concentration. Correlation between DOC concentrations and proportions of A) 22:6n-3, B) 18:3n-3, C) 20:5n-3, D) 16:1n-7, and E) 20:4n-6, which were fatty acids identified to contribute most to observed difference between composition of perch caught in clear- and brown-water lakes. Spearman’s rank correlation coefficient (r_s_) is shown. ** *P*<0.005; n.s. = not significant.

SIMPER identified five PUFAs that contributed together to 55.3% of separation between perch caught in clear- and brown-water lakes: 22:6n-3 (16.6% contribution), 18:3n-3 (11.9% contribution), 20:5n-3 (10.7% contribution), 16:1n-7 (8.7% contribution), and 20:4n-6 (7.3% contribution) (Fig 4; S1 File). As the five identified FA corresponded to different axes in ordination, we refrained from pooling them and instead used individual correlations to investigate the connection between FA proportions and DOC concentrations. In general, a slight trend of a decrease in 22:6n-3, 20:5n-3, 18:3n-3, and 20:4n-6 with increasing DOC concentrations was observed (Fig 5). However, Spearman’s rank correlation were not significant between proportions of respective FA in littoral and pelagic perch and DOC, except for a negative correlation of 20:5n-3 in pelagic fish (r_s_ = -0.669; *P* < 0.0001). Also, there was a significant positive correlation between proportions of 16:1n-7 and DOC concentrations in pelagic perch (r_s_ = 0.606; *P* < 0.0001), but the correlation was not significant in littoral perch.

### Laboratory experiment

**Fig 6:**
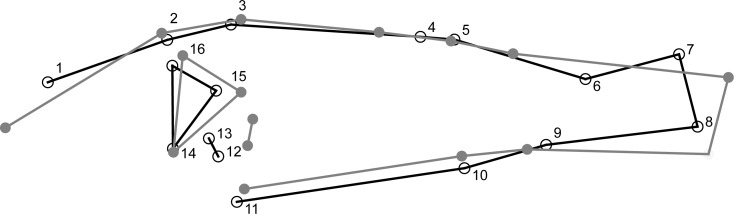
Results of shape analyses of perch from the laboratory experiment. Shape differences between phenotypes induced by habitat structure and feeding mode in the laboratory experiment. Phenotypes of the littoral and pelagic treatments are depicted black and grey lines respectively. Shape-change outlines of Discriminate Function Analysis are magnified ten-fold.

Habitat structure and feeding mode altered the morphology of young-of-the-year fish within 8 weeks. Albeit DFA indicated no significant difference between the phenotypes of the different treatments, 80% of the individuals induced by littoral or pelagic treatment respectively were assigned to the correct group and a change of morphology was visible (Fig 6).

**Fig 7:**
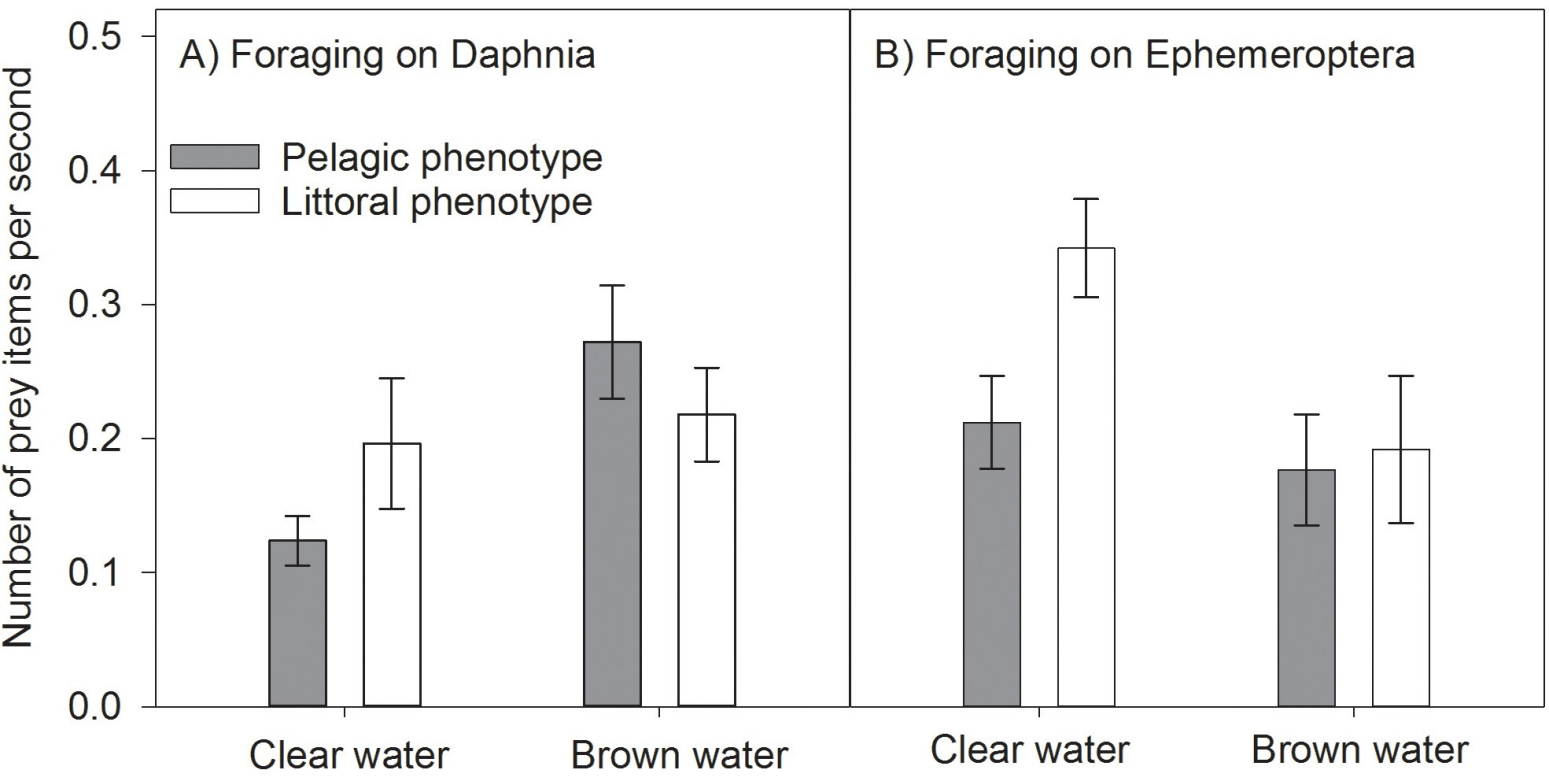
Graphical representation of results from experiment on perch foraging efficiency. Results of A) foraging on *Daphnia*, representing pelagic prey and B) foraging on Ephemeroptera as littoral prey of perch phenotypes (pelagic and littoral) under different visual conditions (clear water, brown water). Average number of prey items captured per second are shown (± SE).

Pelagic phenotypes of perch were more efficient in feeding on *Daphnia* in brown water than in clear water, whereas there was no difference for littoral phenotypes (Table 3, Fig 7). We found a significant interaction of the factor phenotype of foraging in clear and brown water with pelagic being more efficient than littoral phenotypes in brown water (Table 3, Fig 7). Perch foraging on Ephemeroptera were overall less efficient in brown water than in clear water (Table 3, Fig 7). In particular, the littoral phenotype was more efficient in foraging on Ephemeroptera in clear water, but in brown water, there was no difference between littoral and pelagic phenotypes (Table 3, Fig 7).

**Table 3:** Results of experiments on foraging efficiency.

Reference category	Contrast	Estimate	Standard error	*t*-value	*P*-value
**A) Foraging on *Daphnia***					
Clear-water and littoral phenotype	pelagic phenotype	-0.06	0.04	-1.28	0.202
	brown-water	0.02	0.05	0.33	0.739
	pelagic phenotype x brown-water	0.13	0.07	2.04	0.041
Brown-water and littoral phenotype	pelagic phenotype	0.08	0.05	1.60	0.110
Brown- water and pelagic phenotype	clear-water	-0.15	0.05	-3.25	0.001
**B) Foraging on Ephemeroptera**					
Clear-water and littoral phenotype	pelagic phenotype	-0.13	0.06	-2.15	0.040
	brown-water	-0.15	0.06	-2.49	0.019
	pelagic phenotype x brown-water	0.11	0.09	1.34	0.190
Brown-water and littoral phenotype	pelagic phenotype	-0.02	0.06	-0.26	0.800
Brown-water and pelagic phenotype	clear-water	0.04	0.06	0.59	0.562

Results of A) generalized mixed model for foraging on pelagic prey (*Daphnia*), and B) linear model for foraging on littoral prey (Ephemeroptera). Effects of induced phenotype (littoral, pelagic) and visual condition (clear water, brown water). Treatment contrasts are shown.

## Discussion

The morphological results from our field study confirms previous results of Bartels et al. [28, 29], showing that population divergence in perch decreases with decreasing water transparency generated by DOC. Increasing DOC concentrations are assumed to restructure aquatic food webs due to light limitation, further affecting the quality of resources via altered fatty acid compositions of prey items [24, 33]. As perch divergence is strongly correlated to growth rate, and growth rate is affected by the prevalence of essential biomolecules, such as PUFAs, we predicted a direct connection between the decrease in the abundance of essential fatty acids in muscle tissue and the decrease in divergence.

Albeit we found a slight decrease with increasing DOC concentrations in the general prevalence of PUFAs between perch caught in clear- and brown water lakes (22:6n-3, 18:3n-3, 20:5n-3, and 20:4n-6), no overall statistically significant relationship was found (except for 20:5n-3 in pelagic fish). Instead, as revealed by our laboratory experiment, foraging efficiency on littoral prey items seems to be affected by browning waters. Therefore, browning water may have a strong effect on the forager’s ability to find particular food resources, resulting in the reduced development of evolutionary traits, such as habitat- specific morphological divergence.

Fatty acid composition between perch caught in clear- and brown-water lakes differed significantly, primarily attributable to differences in proportions of 22:6 n-3, 18:3n-3, 20:5n-3, and 20:4n-6, with higher proportions found in pelagic perch. In the study of Happel et al. [62] 22:6n-3, 20:5n-3, and 20:4n-6 were also found to be important pelagic indicators in yellow perch (*Perca flavescens*) of Lake Michigan, USA. These long-chain fatty acids are considered to be the physiologically active forms of n-3 and n-6 PUFAs, which are essential compounds of the vertebrate cell membranes, and are important for the growth and development of fish [32, 63, 64]. To maintain an optimal physiological status, it is necessary that fish take up these essential compounds via their diet [31].

In a previous study, we showed differences between pelagic and littoral resources of the clear water lakes presented herein (i.e. Ljustjärn, Långsjön, and Erken; [52]). We found that n-3 PUFAs, such as 22:6n-3, 18:3n-3, and 18:4n-3 are highly abundant in copepoda and especially in cladocera from the pelagic zones, whereas littoral macroinvertebrate composition was characterized by higher proportions of fatty acids with fewer double bonds, e.g.16:1n-7, 16:0, 18:1n-9, 18:1n-7, and 18:2n-6 [52]. These fatty acids are prevalent in littoral macroinvertebrates [41, 65], and may serve as littoral biomarkers, indicating for example contributions from terrestrial detritus (18:2n-6) [66].

We were not able to connect an overall decrease in the abundance of our target fatty acids that were mainly responsible for differences between perch caught in clear- and brown-water lakes and increasing DOC. Nevertheless, we found a significant connection between increasing DOC concentration and decreasing 20:5n-3 concentrations in pelagic phenotypes. Unfortunately, our dataset did not allow correlation analyses of fatty acid composition of resources with increasing DOC concentrations. Further investigation is needed to resolve the underlying mechanisms of these findings. Potentially, the alteration in fatty acid composition in perch might be due to an overall change in the phytoplankton community. In many brown water lakes, the flagellate *Gonyostomum semen*, has been found to be very common [67]. Although it is rich in 20:5n-3 [68], trophic transfer of fatty acids via zooplankton grazing may be limited due to the large size of this organism [69]. Alternatively, an overall change in zooplankton community composition, towards a decrease of abundance of EPA rich cladocera [70, 71] could be driven by high DOC levels. This conclusion is supported by the findings of Bartels et al. [29] that the dietary contribution of cladocera is a key predictor of population divergence in perch, with lower proportions in high DOC lakes.

In the studied lakes, concentrations of DOC and TP were highly correlated and confounding effects on fatty acid composition cannot be completely ruled out, as we did not investigate potential changes in phytoplankton communities. For example, Müller-Navarra et al. [72] found a decrease of specific n-3 PUFAs with increasing trophic state due to an overall shift in phytoplankton community towards low-quality species, such as cyanobacteria. However, lakes included in their study ranged in TP-concentrations up to 230 μg l ^-1^, which by far exceeds the concentrations of the lakes we studied herein. Contrary to these results, Lau et al. [41] found a general increase of n-3-FAs with increasing trophic levels in the littoral invertebrate *Asellus asellus*, suggesting that benthic trophic transfer of FAs gets more efficient as nutrient levels increase.

Our results indicate that essential FAs are not restricting divergence of perch. However, caution should be required when interpreting our results as evidence can be found that consumers may remain rather homeostatic in their FA composition under resource limitation in order to maintain physiological processes [73, 74]. Furthermore, perch might, to a certain degree, be able to adapt to low quality resources by converting fatty acids into longer chains by enzymatic reactions [64, 75, 76]. In contrast, the factor lake contributed the highest explanation of FA variation in perch (26.55%), indicating a multitude of factors involved in FA variation. Further field-based research is needed to unravel the degree of possible variation of FA composition in teleost fish.

A possible explanation to the lack of consistent patterns in FA composition and phenotypic divergence could be related to a strong correlation between prey selection and phenotypic variation. The results of our experiments on the foraging efficiency of perch showed a strong decrease in feeding rate on specific prey items under deteriorated visual conditions. These findings are in line with Jönsson et al. [47] who found reduced encounter rates of pike in humic waters. In contrast to turbid water induced by suspended algae that scatters light, brown water from DOC will absorb the light, resulting in darker images [47]. This may affect detection of particular prey items differently, which occurred in our experiment with a higher foraging rate of *Daphnia* compared to Ephemeroptera in brown water. Similarly, Ranåker et al. [77] showed a change of selectivity of pikeperch (*Sander lucioperca*) for specific prey species (from perch to roach *Rutilus rutilus*) under degraded optical conditions due to different predator avoidance tactics. Motion of prey items might be an important factor in prey detection, as suggested by Bartels et al. [29]. In their field study, they found lower proportions of cladocera in stomachs of pelagic perch from low transparency lakes, whereas contributions of that fast and irregular moving copepoda increased. Although we did not use copepods, but rather slowly moving cladocera in our experiment an even stronger foraging efficiency on faster moving prey such as copepoda can be expected based on the findings of Bartels et al. [29].

Interestingly, we found a significant positive correlation of 16:1n-7 and DOC in pelagic perch.

Although 16:1 n-7 is widely used as diatom biomarker, high proportions of this FA have also been found in anaerobic photosynthetic bacteria, such as sulphur- or iron-reducing bacteria and high concentrations of these compounds were also found in sediments of anoxic lakes [78–80]. These bacteria can strive in anoxic zones of DOC rich lakes that resulting from light attenuation [81, 82]. Furthermore, we found a significant correlation of DOC and a number of fatty acids that we pooled into the group of bacterial fatty acids in pelagic phenotypes (S1 Fig). Biosynthesis of heterotrophic bacteria produces iso- and anteiso- branched as well as odd chained saturated fatty acids, that can be used a tracers [83]. Therefore, our results are in accordance with previous studies indicating an increasing contribution of heterotrophic bacteria to lake food webs due to increasing DOC [23, 34]. However, to our knowledge, this study is the first one to directly trace this change of the trophic base of the food web up to the higher trophic levels. This result suggests that DOC input promotes longer pelagic food chains via the microbial loop, which includes heterotrophic bacteria, flagellates, zooplankton and fish [18, 23]. Longer food chains with increased numbers of trophic links will further result in an overall lower trophic transfer efficiency which may constrain production at higher trophic levels [18, 24, 84]. The limitation of energy, i.e. the quantity of resources available for perch growth can serve as an alternative explanation for the decrease of population divergence of perch in browning waters.

The shift of littoral perch from a diet based on benthic invertebrates towards a diet dominated by zooplankton with increasing DOC-concentrations may have cascading effects on the lower trophic levels with subsequent consequences for the whole aquatic ecosystem dynamics and even greenhouse gas emissions [85, 86]. Therefore, our study highlight the ability of terrestrial organic carbon to restructure aquatic ecosystems. So far, this effects was primarily related to altered bottom-up effects via changes in primary production through light limitation (sensu [18]), but our study further demonstrate potential top-down effects through altered prey detection of consumers. Together, our results indicate potential changes in food web interactions that are associated with the widespread browning of freshwaters due to increased inputs of terrestrial carbon [15, 87]. The browning phenomenon has the ability to diminish habitat heterogeneity that generate diversity in evolutionary traits, such as morphological divergence in Eurasian perch. Ultimately, to maintain species diversity it is critical to understand the ecological processes leading to the overall rerouting of energy flows in aquatic food webs due to the degraded optical conditions.

## Supporting Information

S1 Fig Bacterial fatty acids in perch. Correlation between proportions of bacterial fatty acids including iso- and anteiso-branched and odd chained fatty acids and DOC concentrations. Spearman’s rank correlation coefficient (r_s_) is shown. **P < 0.005; n.s. = not significant.
(TIF)

S1 File 3D nMDS of fatty acid composition. Animation of three dimensions of non-metric multidimensional scaling (nMDS) of fatty acid composition (%) from perch caught in the littoral and pelagic zone of the six lakes surveyed. Color shading illustrates the gradient of DOC from light blue = low DOC to dark brown = high DOC. Relative length of vectors from fatty acids identified to contribute most to observed difference in composition between perch caught in clear-and brown water lakes depict strength in positioning in the respective dimension.
(MP4)

S1 Table **PERMANOVA results of separate analyses of fatty acids of perch in A) brown (Oppsveten, Strandsjön, and Fälaren), and B) clear-water (Ljustjärn, Långsjön, and Erken) lakes.** Comparisons were based on arcsine-square root transformed fatty acids (% fatty acids) and on ordination of Euclidean distance matrices. Differences of FA between lakes, habitats (nested within lake), and total length (as a covariate) were tested. Significance of analyses was conducted on permutation of residuals under a reduced model (9999 permutations) with type I sums of squares. Proportion of variance explained was calculated from sums of squares.
(DOCX)

## References

[1] Parmesan C. Ecological and evolutionary responses to recent climate change. Annual Review of Ecology Evolution and Systematics. 2006;37:637–69. 10.1146/annurev.ecolsys.37.091305.110100 .

[2] Dall SRX, Bell AM, Bolnick DI, Ratnieks FLW. An evolutionary ecology of individual differences. Ecology Letters. 2012;15(10):1189–98. 10.1111/j.1461-0248.2012.01846.x . 22897772 PMC3962499

[3] Rueffler C, Van Dooren TJM, Leimar O, Abrams PA. Disruptive selection and then what? Trends in Ecology & Evolution. 2006;21(5):238–45. 10.1016/j.tree.2006.03.003 . 16697909

[4] Hendry AP. Ecological speciation! Or the lack thereof? Canadian Journal of Fisheries and Aquatic Sciences. 2009;66(8):1383–98. 10.1139/f09-074 .

[5] Streelman JT, Danley PD. The stages of vertebrate evolutionary radiation. Trends in Ecology & Evolution. 2003;18(3):126–31. .

[6] Smith TB, Skúlason S. Evolutionary significance of resource polymorphisms in fishes, amphibians, and birds. Annual Review of Ecology and Systematics. 1996;27:111–33.

[7] Puebla O. Ecological speciation in marine v. freshwater fishes. Journal of Fish Biology. 2009;75(5):960–96. 10.1111/j.1095-8649.2009.02358.x . 20738594

[8] Räsänen K, Hendry AP. Disentangling interactions between adaptive divergence and gene flow when ecology drives diversification. Ecology Letters. 2008;11(6):624–36. 10.1111/j.1461-0248.2008.01176.x . 18384363

[9] Wagner CE, Harmon LJ, Seehausen O. Ecological opportunity and sexual selection together predict adaptive radiation. Nature. 2012;487(7407):366–U124. 10.1038/nature11144 . 22722840

[10] Seehausen O. Conservation: Losing biodiversity by reverse speciation. Current Biology. 2006;16(9):R334–R7. 10.1016/j.cub.2006.03.080 . 16682344

[11] Vonlanthen P, Bittner D, Hudson AG, Young KA, Muller R, Lundsgaard-Hansen B, et al Eutrophication causes speciation reversal in whitefish adaptive radiations. Nature. 2012;482(7385):357–U1500. 10.1038/nature10824 . 22337055

[12] Revenga C, Campbell I, Abell R, de Villiers P, Bryer M. Prospects for monitoring freshwater ecosystems towards the 2010 targets. Philos Trans R Soc B-Biol Sci. 2005;360(1454):397–413. 10.1098/rstb.2004.1595 . PMC1569454 15814353

[13] Schindler DE, Vallentyne JR. The algal bowl: overfertilization of the world's freshwaters and estuaries Edmonton, Alberta, Canada: University of Alberta Press; 2008.

[14] Wood PJ, Armitage PD. Biological effects of fine sediment in the lotic environment. Environ Manage. 1997;21(2):203–17. 10.1007/s002679900019 . 9008071

[15] Roulet N, Moore TR. Environmental chemistry—Browning the waters. Nature. 2006;444(7117):283–4. 10.1038/444283a . 17108948

[16] Zhang J, Hudson J, Neal R, Sereda J, Clair T, Turner M, et al Long-term patterns of dissolved organic carbon in lakes across eastern Canada: Evidence of a pronounced climate effect. Limnology and Oceanography. 2010;55(1):30–42. 10.4319/lo.2010.55.1.0030 .

[17] Solomon CT, Jones SE, Weidel BC, Buffam I, Fork ML, Karlsson J, et al Ecosystem consequences of changing inputs of terrestrial dissolved organic matter to lakes: current knowledge and future challenges. Ecosystems. 2015;18(3):376–89. 10.1007/s10021-015-9848-y

[18] Karlsson J, Byström P, Ask J, Ask P, Persson L, Jansson M. Light limitation of nutrient-poor lake ecosystems. Nature. 2009;460(7254):506–U80. 10.1038/nature08179 . 19626113

[19] Keller W, Heneberry J, Leduc J, Gunn J, Yan N. Variations in epilimnion thickness in small Boreal Shield Lakes: Relationships with transparency, weather and acidification. Environ Monit Assess. 2006;115(1–3):419–31. 10.1007/s10661-006-7237-x . 16614780

[20] Hilt S, Köhler J, Adrian R, Monaghan MT, Sayer CD. Clear, crashing, turbid and back—long-term changes in macrophyte assemblages in a shallow lake. Freshwater Biology. 2013;58(10):2027–36. 10.1111/fwb.12188 .

[21] Vadeboncoeur Y, Jeppesen E, Vander Zanden MJ, Schierup HH, Christoffersen K, Lodge DM. From Greenland to green lakes: Cultural eutrophication and the loss of benthic pathways in lakes. Limnology and Oceanography. 2003;48(4):1408–18. .

[22] Algesten G, Sobek S, Bergström AK, Agren A, Tranvik LJ, Jansson M. Role of lakes for organic carbon cycling in the boreal zone. Global Change Biology. 2004;10(1):141–7. 10.1111/j.1365-2486.2003.00721.x .

[23] Ask J, Karlsson J, Persson L, Ask P, Byström P, Jansson M. Terrestrial organic matter and light penetration: Effects on bacterial and primary production in lakes. Limnology and Oceanography. 2009;54(6):2034–40. 10.4319/lo.2009.54.6.2034 .

[24] Finstad AG, Helland IP, Ugedal O, Hesthagen T, Hessen DO. Unimodal response of fish yield to dissolved organic carbon. Ecology Letters. 2013: 10.1111/ele.12201 24165396

[25] Karlsson J, Bergstrom AK, Bystrom P, Gudasz C, Rodriguez P, Hein C. Terrestrial organic matter input suppresses biomass production in lake ecosystems. Ecology. 2015;96(11):2870–6. 10.1890/15-0515.1 . 27070007

[26] Svanbäck R, Eklöv P. Effects of habitat and food resources on morphology and ontogenetic growth trajectories in perch. Oecologia. 2002;131(1):61–70. 28547511 10.1007/s00442-001-0861-9

[27] Svanbäck R, Eklöv P. Morphology dependent foraging efficiency in perch: a trade-off for ecological specialization? Oikos. 2003;102(2):273–84. 10.1034/j.1600-0706.2003.12657.x .

[28] Bartels P, Hirsch PE, Svanbäck R, Eklöv P. Dissolved organic carbon reduced habitat coupling by top predators in lake ecosystems. Ecosystems. 19(6):955–967. 10.1007/s10021-016-9978-x

[29] Bartels P, Hirsch PE, Svanbäck R, Eklöv P. Water transparency drives intra-population divergence in Eurasian perch (*Perca fluviatilis*). PLoS One. 2012;7(8):e43641 10.1371/journal.pone.0043641 . 22912895 PMC3422328

[30] Vrede T, Drakare S, Eklöv P, Hein A, Liess A, Olsson J, et al Ecological stoichiometry of Eurasian perch—intraspecific variation due to size, habitat and diet. Oikos. 2011;120(6):886–96. 10.1111/j.1600-0706.2010.18939.x .

[31] Yang XW, Dick TA. Arctic charr (*Salvelinus alpinus*) and rainbow trout (*Oncorhynchus mykiss*) differ in their growth and lipid metabolism in response to dietary polyunsaturated fatty acids. Canadian Journal of Fisheries and Aquatic Sciences. 1994;51(6):1391–400. 10.1139/f94-139 .

[32] Sargent J, McEvoy L, Estevez A, Bell G, Bell M, Henderson J, et al Lipid nutrition of marine fish during early development: current status and future directions. Aquaculture. 1999;179(1–4):217–29. 10.1016/s0044-8486(99)00191-x .

[33] Stasko AD, Gunn JM, Johnston TA. Role of ambient light in structuring north-temperate fish communities: potential effects of increasing dissolved organic carbon concentration with a changing climate. Environ Rev. 2012;20(3):173–90. 10.1139/a2012-010 .

[34] Faithfull CL, Bergström AK, Vrede T. Effects of nutrients and physical lake characteristics on bacterial and phytoplankton production: A meta-analysis. Limnology and Oceanography. 2011;56(5):1703–13. 10.4319/lo.2011.56.5.1703 .

[35] Lepistö L, Rosenström U. The most typical phytoplankton taxa in four types of boreal lakes. Hydrobiologia. 1998;370:89–97. 10.1023/a:1017014330045 .

[36] Hiltunen M, Strandberg U, Taipale SJ, Kankaala P. Taxonomic identity and phytoplankton diet affect fatty acid composition of zooplankton in large lakes with differing dissolved organic carbon concentration. Limnology and Oceanography. 2015;60(1):303–17. 10.1002/lno.10028 .

[37] Strandberg U, Taipale SJ, Hiltunen M, Galloway AWE, Brett MT, Kankaala P. Inferring phytoplankton community composition with a fatty acid mixing model. Ecosphere. 2015;6(1):18 10.1890/es14-00382.1 .

[38] Galloway AWE, Taipale SJ, Hiltunen M, Peltomaa E, Strandberg U, Brett MT, et al Diet-specific biomarkers show that high-quality phytoplankton fuels herbivorous zooplankton in large boreal lakes. Freshwater Biology. 2014;59(9):1902–15. 10.1111/fwb.12394 .

[39] Brett MT, Müller-Navarra DC. The role of highly unsaturated fatty acids in aquatic food web processes. Freshwater Biology. 1997;38(3):483–99. 10.1046/j.1365-2427.1997.00220.x .

[40] Parrish CC. Determination of total lipid, lipid classes, and fatty acids in aquatic samples In: Arts MT, Wainman BB, editors. Lipids in Freshwater Ecosystems: Springer, New York; 1999 p. 4–20.

[41] Lau DCP, Goedkoop W, Vrede T. Cross-ecosystem differences in lipid composition and growth limitation of a benthic generalist consumer. Limnology and Oceanography. 2013;58(4):1149–64. 10.4319/lo.2013.58.4.1149 .

[42] Volk C, Kiffney P. Comparison of fatty acids and elemental nutrients in periphyton, invertebrates, and cutthroat trout (*Oncorhynchus clarki*) in conifer and alder streams of western Washington state. Aquatic Ecology. 2012;46(1):85–99. 10.1007/s10452-011-9383-7 .

[43] Heintz RA, Nelson BD, Hudson J, Larsen M, Holland L, Wipfli M. Marine subsidies in freshwater: Effects of salmon carcasses on lipid class and fatty acid composition of juvenile coho salmon. Transactions of the American Fisheries Society. 2004;133(3):559–67. 10.1577/t03-035.1 .

[44] Litzow MA, Bailey KM, Prahl FG, Heintz R. Climate regime shifts and reorganization of fish communities: the essential fatty acid limitation hypothesis. Mar Ecol-Prog Ser. 2006;315:1–11. 10.3354/meps315001 .

[45] Estlander S, Nurminen L, Olin M, Vinni M, Immonen S, Rask M, et al Diet shifts and food selection of perch *Perca fluviatilis* and roach *Rutilus rutilus* in humic lakes of varying water colour. Journal of Fish Biology. 2010;77(1):241–56. 10.1111/j.1095-8649.2010.02682.x . 20646150

[46] Ranåker L, Jönsson M, Nilsson PA, Brönmark C. Effects of brown and turbid water on piscivore-prey fish interactions along a visibility gradient. Freshwater Biology. 2012;57(9):1761–8. 10.1111/j.1365-2427.2012.02836.x .

[47] Jönsson M, Ranåker L, Nilsson PA, Brönmark C. Foraging efficiency and prey selectivity in a visual predator: differntial effects of turbid and humic waters. Canadian Journal of Fisheries and Aquatic Sciences. 2013;70:1685–90.

[48] Abrahams M, Kattenfeld M. The role of turbidity as a constraint on predator-prey interactions in aquatic environments. Behav Ecol Sociobiol. 1997;40(3):169–74. 10.1007/s002650050330 .

[49] Shoup DE, Wahl DH. The effects of turbidity on prey selection by piscivorous Largemouth bass. Transactions of the American Fisheries Society. 2009;138(5):1018–27. 10.1577/t09-015.1 .

[50] Persson L. Effects of reduced interspecific competition on resource utilization in perch (*Perca fluviatilis*). Ecology. 1986;67(2):355–64. 10.2307/1938578 .

[51] Bookstein FL. Morphometric tools for landmark data New York: Cambridge University Press; 1991.

[52] Scharnweber K, Strandberg U, Marklund MHK, Eklöv P. Combining resource use assessment techniques reveal trade-offs in trophic specialization of polymorphic perch. Ecosphere. 2016;7(8):e01387 10.1002/ecs2.1387

[53] Klingenberg CP. MorphoJ: an integrated software package for geometric morphometrics. Molecular Ecology Resources. 2011;11(2):353–7. 10.1111/j.1755-0998.2010.02924.x 21429143

[54] Klingenberg CP. Size, shape, and form: concepts of allometry in geometric morphometrics. Dev Genes Evol. 2016;226(3):113–37. 10.1007/s00427-016-0539-2 . 27038023 PMC4896994

[55] Anderson MJ, Gorley RN, Clarke KR. PERMANOVA+ for PRIMER: guide to software and statistical methods Primer-E Ltd 2008.

[56] Olsson J, Eklöv P. Habitat structure, feeding mode and morphological reversibility: factors influencing phenotypic plasticity in perch. Evolutionary Ecology Research. 2005;7(8):1109–23. .

[57] Eklöv P. Group foraging versus solitary foraging efficiency in piscivorous predators—the perch, *Perca fluviatilis*, and pike, *Esox lucius*, patterns. Animal Behaviour. 1992;44(2):313–26. 10.1016/0003-3472(92)90037-a .

[58] R Core Team. R: A language and environment for statistical computing R foundation for statistical Computing, Vienna, Austria 2016.

[59] Bates D, Maechler M, Bolker B, Walker S. lme4: linear mixed-effect models using Eigen and S4. R package version 1.1–9. https://CRANR-projectorg/package=lme4. 2015.

[60] Bolker BM, Brooks ME, Clark CJ, Geange SW, Poulsen JR, Stevens MHH, et al Generalized linear mixed models: a practical guide for ecology and evolution. Trends in Ecology & Evolution. 2009;24(3):127–35. 10.1016/j.tree.2008.10.008 . 19185386

[61] Mirman D. Crowth curve analysis and visualization using R 1st ed. Boca Raton, FL, USA: CRC Press; 2016.

[62] Happel A, Creque S, Rinchard J, Höök T, Bootsma H, Janssen J, et al Exploring yellow perch diets in Lake Michigan through stomach content, fatty acids, and stable isotope ratios. J Gt Lakes Res. 2015;41(3):172–8.

[63] Bell JG, Sargent JR. Arachidonic acid in aquaculture feeds: current status and future opportunities. Aquaculture. 2003;218(1–4):491–9. 10.1016/s0044-8486(02)00370-8 .

[64] Tocher DR. Metabolism and functions of lipids and fatty acids in teleost fish. Reviews in Fisheries Sciences. 2003;11(2):107–84.

[65] Lau DCP, Vrede T, Pickova J, Goedkoop W. Fatty acid composition of consumers in boreal lakes—variation across species, space and time. Freshwater Biology. 2012;57(1):24–38. 10.1111/j.1365-2427.2011.02690.x .

[66] Kelly JR, Scheibling RE. Fatty acids as dietary tracers in benthic food webs. Mar Ecol-Prog Ser. 2012;446:1–22. 10.3354/meps09559 .

[67] Rengefors K, Weyhenmeyer GA, Bloch I. Temperature as a driver for the expansion of the microalga *Gonyostomum semen* in Swedish lakes. Harmful Algae. 2012;18:65–73. 10.1016/j.hal.2012.04.005 .

[68] Gutseit K, Berglund O, Graneli W. Essential fatty acids and phosphorus in seston from lakes with contrasting terrestrial dissolved organic carbon content. Freshwater Biology. 2007;52(1):28–38. 10.1111/j.1365-2427.2006.01668.x .

[69] Lebret K, Fernández MF, Hagman CHC, Rengefors K, Hansson LA. Grazing resistance allows bloom formation and may explain invasion success of *Gonyostomum semen*. Limnology and Oceanography. 2012;57(3):727–34. 10.4319/lo.2012.57.3.0727 .

[70] Persson J, Vrede T. Polyunsaturated fatty acids in zooplankton: variation due to taxonomy and trophic position. Freshwater Biology. 2006;51(5):887–900. 10.1111/j.1365-2427.2006.01540.x .

[71] Ravet JL, Brett MT, Arhonditsis GB. The effects of seston lipids on zooplankton fatty acid composition in Lake Washington, Washington, USA. Ecology. 2010;91(1):180–90. 10.1890/08-2037.1 . 20380207

[72] Müller-Navarra DC, Brett MT, Park S, Chandra S, Ballantyne AP, Zorita E, et al Unsaturated fatty acid content in seston and tropho-dynamic coupling in lakes. Nature. 2004;427(6969):69–72. 10.1038/nature02210 . 14702086

[73] Müller-Navarra DC. The nutritional importance of polyunsaturated fatty acids and their use as trophic markers for herbivorous zooplankton: Does it contradict? Archiv Fur Hydrobiologie. 2006;167(1–4):501–13. 10.1127/0003-9136/2006/0167-0501 .

[74] Frost PC, Tank SE, Turner MA, Elser JJ. Elemental composition of littoral invertebrates from oligotrophic and eutrophic Canadian lakes. Journal of the North American Benthological Society. 2003;22(1):51–62.

[75] Xu XL, Kestemont P. Lipid metabolism and FA composition in tissues of Eurasian perch *Perca fluviatilis* as influenced by dietary fats. Lipids. 2002;37(3):297–304. 10.1007/s11745-002-0894-2 . 11942481

[76] Murray DS, Hager H, Tocher DR, Kainz MJ. Effect of partial replacement of dietary fish meal and oil by pumpkin kernel cake and rapeseed oil on fatty acid composition and metabolism in Arctic charr (*Salvelinus alpinus*). Aquaculture. 2014;431:85–91. 10.1016/j.aquaculture.2014.03.039 .

[77] Ranåker L, Persson J, Jönsson M, Nilsson PA, Brönmark C. Piscivore-prey fish interactions: mechanisms behind diurnal patterns in prey selectivity in brown and clear water. PLoS One. 2014;9(11):8 10.1371/journal.pone.0102002 . PMC4224368 25379665

[78] Napolitano GE. Fatty acids as a trophic and chemical markers in freshwater ecosystems In: Arts MT, Wainman BB, editors. Lipids in Freshwater ecosystems. New York: Springer; 1999 p. 21–44.

[79] Petrišič MG, Ogrinc N. Lipid biomarkers of suspended particulate organic matter in Lake Bled (NW Slovenia). Geomicrobiol J. 2013;30(4):291–301. 10.1080/01490451.2012.688789 .

[80] Fredrickson HL, Cappenberg TE, Deleeuw JW. Polar lipid ester-linked fatty acid composition of Lake Vechten seston- an ecological application of lipid analysis. FEMS Microbiol Ecol. 1986;38(6):381–96. 10.1111/j.1574-6968.1986.tb01751.x .

[81] Craig N, Jones SE, Weidel B, Solomon C. Habitat, not resource availability, limits consumer production in lake ecosystems. Limnology and Oceanography. 2015;60:2079–89.

[82] Brothers S, Köhler J, Attermeyer K, Grossart HP, Mehner T, Meyer N, et al A feedback loop links brownification and anoxia in a temperate, shallow lake. Limnology and Oceanography. 2014;59(4):1388–98. 10.4319/lo.2014.59.4.1388 .

[83] Dalsgaard J, St John M, Kattner G, Müller-Navarra D, Hagen W. Fatty acid trophic markers in the pelagic marine environment. Advances in Marine Biology, Vol 46. 2003;46:225–340. 10.1016/s0065-2881(03)46005-7 . 14601414

[84] Jones RI. The influence of humic substances on lacustrine planktonic food-chains. Hydrobiologia. 1992;229:73–91.

[85] Atwood TB, Hammill E, Greig HS, Kratina P, Shurin JB, Srivastava DS, et al Predator-induced reduction of freshwater carbon dioxide emissions. Nature Geoscience. 2013;6(3):191–4. 10.1038/ngeo1734 .

[86] Schindler DE, Carpenter SR, Cole JJ, Kitchell JF, Pace ML. Influence of food web structure on carbon exchange between lakes and the atmosphere. Science. 1997;277(5323):248–51. 10.1126/science.277.5323.248 .

[87] Monteith DT, Stoddard JL, Evans CD, de Wit HA, Forsius M, Hogasen T, et al Dissolved organic carbon trends resulting from changes in atmospheric deposition chemistry. Nature. 2007;450(7169):537–U9. 10.1038/nature06316 . 18033294

