# Supplementary figures and images for: Decrease of Population Divergence in Eurasian Perch (Perca fluviatilis) in Browning Waters: Role of Fatty Acids and Foraging Efficiency
Source: PLoS One. 2016 Sep 9;11(9):e0162470. doi: 10.1371/journal.pone.0162470 (PMC5017650; doi:10.1371/journal.pone.0162470)

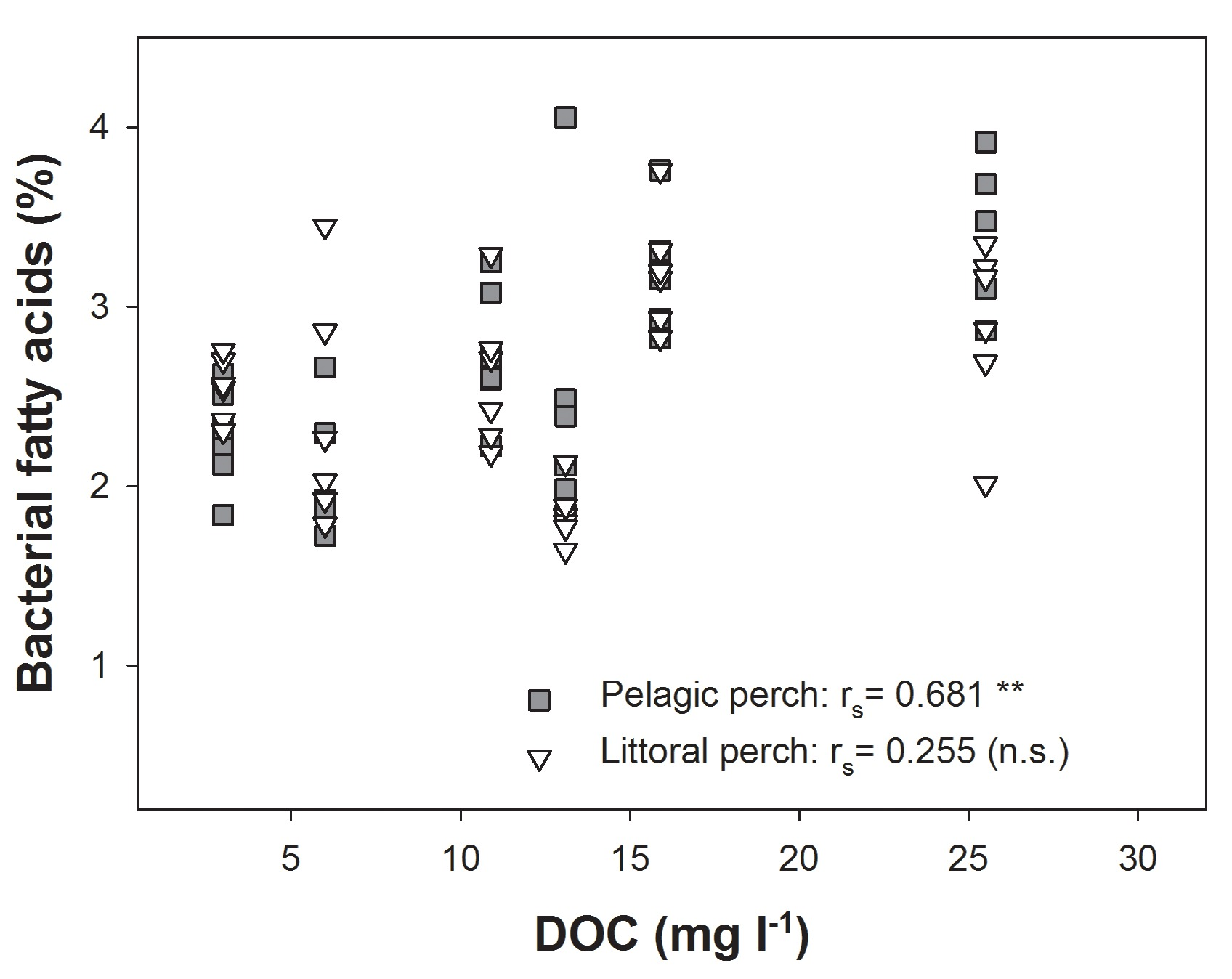

Supplement: S1 Fig — Correlation between proportions of bacterial fatty acids including iso- and anteiso-branched and odd chained fatty acids and DOC concentrations. Spearman’s rank correlation coefficient (rs) is shown. **P < 0.005; n.s. = not significant. (TIF) [file pone.0162470.s001.tif]
