# Supplementary material for: Decrease of Population Divergence in Eurasian Perch (Perca fluviatilis) in Browning Waters: Role of Fatty Acids and Foraging Efficiency
Source: PLoS One. 2016 Sep 9;11(9):e0162470. doi: 10.1371/journal.pone.0162470 (PMC5017650; doi:10.1371/journal.pone.0162470)
Supplement: S1 Table — PERMANOVA results of separate analyses of fatty acids of perch in A) brown (Oppsveten, Strandsjön, and Fälaren), and B) clear-water (Ljustjärn, Långsjön, and Erken) lakes. Comparisons were based on arcsine-square root transformed fatty acids (% fatty acids) and on ordination of Euclidean distance matrices. Differences of FA between lakes, habitats (nested within lake), and total length (as a covariate) were tested. Significance of analyses was conducted on permutation of residuals under a reduced model (9999 permutations) with type I sums of squares. Proportion of variance explained was calculated from sums of squares. (DOCX) [file pone.0162470.s003.docx]

**S1 Table. PERMANOVA results of separate analyses of fatty acids of perch in A) brown (Oppsveten, Strandsjön, and Fälaren), and B) clear-water (Ljustjärn, Långsjön, and Erken) lakes.** Comparisons were based on arcsine-square root transformed fatty acids (% fatty acids) and on ordination of Euclidean distance matrices. Differences of FA between lakes, habitats (nested within lake), and total length (as a covariate) were tested. Significance of analyses was conducted on permutation of residuals under a reduced model (9999 permutations) with type I sums of squares. Proportion of variance explained was calculated from sums of squares.

|  | **Factor** | **Pseudo-*F*** | ***P*-value** | **% variance explained** |
| --- | --- | --- | --- | --- |
| **A) Brown-water lakes** | lake | 4.98 | 0.0001 | 20.2 |
|  | habitat (lake) | 2.01 | 0.0214 | 12.2 |
|  | total length | 4.26 | 0.0032 | 8.6 |
| **B) Clear-water lakes** |  |  |  |  |
|  | lake | 10.46 | 0.0001 | 27.7 |
|  | habitat (lake) | 3.67 | 0.0002 | 14.5 |
|  | total length | 14.73 | 0.0001 | 19.5 |
